# Inclusion under the Mental Capacity Act (2005): A review of research policy guidance and governance structures in England and Wales

**DOI:** 10.1111/hex.13165

**Published:** 2020-11-27

**Authors:** Hayley Ryan, Rob Heywood, Oluseyi Jimoh, Anne Killett, Peter E Langdon, Ciara Shiggins, Karen Bunning

**Affiliations:** ^1^ University of East Anglia England UK; ^2^ University of Warwick England UK; ^3^ Centre of Research Excellence in Aphasia Recovery and Rehabilitation Melbourne Australia; ^4^ School of Allied Health Human Services and Sport La Trobe University Melbourne Australia

**Keywords:** capacity, Code of Practice, communication, consent, ethics, Mental Capacity Act

## Abstract

**Objective:**

To investigate how people with communication and understanding difficulties, associated with conditions such as dementia, autism and intellectual disability, are represented in research guidance supplementary to the Mental Capacity Act (MCA: 2005) in England and Wales.

**Methods:**

A documentary survey was conducted. The sample comprised the MCA Code of Practice (CoP: 2007) and 14 multi‐authored advisory documents that were publicly available on the Health Research Authority website. Textual review of key words was conducted followed by summative content analysis.

**Results:**

Representation of people with communication and understanding difficulties was confined to procedural information and position statements that focused mainly on risk management and protection. Whilst a need to engage potential participants was recognized, guidance provided was imprecise.

**Conclusions:**

Tensions exist between the protection versus empowerment of people with communication and understanding difficulties in research. The development of structured, evidence‐based guidance is indicated.

**Patient or public contribution:**

People with communication and understanding difficulties and carers participated in a working group to explore, discuss and interpret the findings.

## 1 INTRODUCTION

Including adults with communication and understanding difficulties in ethically sound research is a complex proposition. In England and Wales, the Mental Capacity Act (MCA, 2005),^1^ a piece of legislation applicable to individuals aged 16 years and above, was introduced primarily to protect vulnerable people who may lack capacity for informed decision‐making.^2^ Separate provisions were enacted for approving intrusive research. Despite these provisions, the under‐representation of people with communication and understanding difficulties in research is widespread.^3^, ^4^ This may affect our understanding of the needs of these groups, and the efficacy of new treatments and interventions. The Research Governance Framework for Health and Social Care in England and Wales^5^ requires that research participants reflect the diversity of the wider population and advises against the routine exclusion of under‐researched groups, including those with disabilities.^6^ Researchers are responsible for ensuring that their research complies with the requirements of the MCA (2005), and that people with capacity‐affecting conditions are included appropriately.^7^

Gaining consent is a fundamental prerequisite for involving human beings in research.^8^ Founded on the principle of respect for autonomy,^9^, ^10^, ^11^ it formally recognizes people's interest in making decisions, acting voluntarily, and understanding and processing appropriate information relating to these decisions. However, certain conditions, such as dementia, autism and intellectual disability, may affect the individual's decision‐making capacity.^12^, ^13^ Another common characteristic of these conditions is communication and understanding difficulties, which may pose challenges and complicate assessments of capacity.^14^, ^15^ People with capacity‐affecting conditions make up a significant proportion of the UK population. The number of people with dementia is projected to rise to 1.6 million by 2040^16^; 1 in 100 people have autism^17^; there are over 1.2 million stroke survivors^18^; and a further 1.5 million people with intellectual disabilities.^19^

The Code of Practice (CoP: 2007) accompanies the MCA (2005) providing guidance for interpreting the legislation.^20^, ^21^ For example, paragraph 11.29 of the CoP (2007) stipulates that the patient's wishes and feelings must be considered regardless of their capacity for deciding about their own research participation. In the context of research, the MCA (2005) has been criticized for lacking an appropriate balance between protection from exploitation and empowerment, with emphasis placed on the former.^22^ Indeed, the MCA post‐legislative scrutiny criticized the implementation of the Act in care contexts, noting a culture of protection and paternalism amongst professionals working with people who may lack capacity.^23^ This appears to be in opposition to the culture of empowerment that has grown in recent years within health and social services in the UK for different care groups, including people with intellectual disabilities,^24^ autism and^25^ dementia.^26^ There is an obvious tension between managing any risks associated with research participation and ensuring individual rights are supported.^5^, ^27^

Assessment of participant capacity (the ability to understand the information relevant to the decision, to retain, to use or weigh it up and to communicate the decision) is a primary requirement in ethical research under the MCA (2005).^1^, ^28^, ^29^ A person's decision is viewed as their own and therefore sacrosanct. However, where a person is lacks capacity, additional requirements of the MCA (2005) need to be satisfied to authorize their participation in research. In such cases, the appointment of a consultee—a person who is able to advise on the individual's likely wishes and desires regarding participation in research—is recommended. The involvement of another person might seem diametrically opposed to any notion of empowerment and might prioritize the consultee's personal views over those of the individual. Furthermore, Jackson^30^ observed that capacity and incapacity do not have clear boundaries and should be viewed on a continuum.^31^

The CoP (2007)^21^ attempts to redress the imbalance between protection and empowerment by recommending consideration of the views of potential participants who lack capacity. This is consistent with empowerment theory, which highlights strengths and capabilities, rather than cataloguing risk factors.^32^, ^33^, ^34^ As a construct, empowerment theory examines interaction of individual competencies, systemic support or facilitation, and proactive behaviour affecting policy development and the social change process.^35^, ^36^ Joining efforts with others to gain access to resources and to achieve goals are considered critical to empowerment. Zimmerman and colleagues identified three components of empowerment: intrapersonal (how people think about their capacity to influence others); interactional (the transactions between people and environments); and behavioural (what is done to influence change in the environment).^35^ Thus, empowerment may be viewed as a complementary process to the protection of rights, but also one that invites support from others.^37^ This is where the concept of assent, an ‘expansive, educational and multimodal’^38^ process that is adaptable to individual needs, becomes relevant. Assent is described as an individual's agreement to participate in research, where consultee affirmation has been established. Mere absence of dissent is not enough to infer assent; it requires that researchers engage with the prospective participant in ways that respond to the individual's communication and understanding needs..^39^ Whilst the CoP (2007) urges consideration of the individual's wishes and preferences, how to evidence them is usually left to local interpretation. Sibley and colleagues drew a distinction between ‘respecting and encouraging a decision’: the former acknowledges individual rights; the latter focuses on the individual engagement process.^40^

The CoP (2007) has been criticized for being lengthy and rarely accessed.^23^ Supplementary research guidance is provided on the Health Research Authority (HRA) website. However, processes used to support decision‐making in everyday life,^41^ for example ‘Supported Decision‐making’^42^; ‘Partnership of Consent Protocol’^43^, ^44^; and ‘Active Support’,^45^ are not formally recognized practices. The national statement on ethical conduct in research recommends that information is presented for optimal accessibility by the person.^46^ For example, simplified language in large print with pictorial support has been used with people who have intellectual disabilities (termed Easy Read)^47^; aphasia‐friendly resources for people post‐stroke,^48^, ^49^ and digital computer technology with people with autism.^50^ According to relevance theory, we naturally engage with information (spoken, written or symbolic) that requires the least cognitive effort for the most successful understanding.^51^ This requires deliberate address of critical aspects of language including its form (syntax and grammar), content (semantics or meanings and vocabulary) and use (intended purpose of the message).^52^

The current study was part of an investigation into the ethico‐legal landscape for the development of an assent‐based process for the inclusion of adults with communication and understanding difficulties in ethically sound research in England and Wales. The aim was to investigate how people with communication and understanding difficulties are considered in the research guidance in England and Wales. This is an international concern for legal systems across the globe.^53^, ^54^ The research question was as follows: How does the CoP (2007), as the operational document of the MCA (2005), and HRA research guidance support the representation of people with communication and understanding difficulties in research?

## 2 METHODS

### 2.1 Design & sample

A documentary survey was conducted. The sample comprised: 1. Research guidance supplementary to the CoP (2007) of potential interest to researchers and publicly available on the HRA website; 2. The CoP (2007) providing operational guidance to the research aspect of the MCA (2005).

#### 2.1.1 HRA research guidance

The HRA website (www.hra.nhs.uk) provides a central repository of information for researchers in England and Wales, which includes supplementary guidance to the MCA (2005) and the CoP (2007). A comprehensive sample of e‐documents (www.hra.nhs.uk, 2019) was established (N = 14). Documents were included if they: contained research guidance supplementary and with reference to the MCA (2005) and/or the CoP (2007); considered mental capacity, informed consent and supporting the inclusion of people with communication and understanding difficulties in research; and were available and accessible in e‐format. Documents that focused on clinical trials, research with children or research in emergency settings were excluded because they are addressed by alternative provisions. Download of the e‐documents resulted in one exclusion due to a broken hyperlink and no other form of access via the HRA (see Appendix A for the list of documents).

#### 2.1.2 The CoP (2007)

The CoP (2007) was retrieved from the UK Government website. Comprising 16 chapters of key provisions under the MCA (2005), analysis focused on Chapter 11 (14 out of 301 total pages), which addresses research and *‘provides guidance on how the Act sets out specific safeguards and controls for research involving, or in relation to, people lacking capacity to consent to their participation.’* [CoP: Page 4]. Text exclusions were as follows: separate provisions for urgent treatment during a research project and research involving human tissue: 11.32‐11.40, for reasons stated previously; and illustrations of the main content to be analysed, for example case vignettes and definitions of key terms.

### 2.2 Data analysis

#### 2.2.1 Word referent frequency

In order to establish how adults with communication and understanding difficulties are represented in research guidance, a survey of surface‐level vocabulary was conducted. Firstly, a list of key words (termed referents) was generated that related to the following domains of interest: communication and understanding difficulties; decision‐making and capacity; and inclusion in research. A first iteration was compiled by each of two researchers independently. Secondly, the researchers met with the Chief Investigator and the two lists were compared. This involved grouping similar words together (eg communication, language and speech) and identifying any differences between the two lists of referents. Once consensus had been reached, a combined list was generated. Thirdly, the list was used to locate word referents and their frequency of occurrence in an electronic search of each document using NVivo‐12 software.

#### 2.2.2 Summative content analysis

Summative content analysis^55^ was carried out on the entire dataset by two researchers working independently. Latent content analysis was conducted to explore meanings.^55^ Based on their homogeneity, meanings were grouped, resulting in a first level of nodes, termed ‘organising themes’. This gave rise to a second level of nodes, termed ‘sub‐themes’. The Chief Investigator (last author) facilitated two meetings at which the assignment of excerpts to organizing themes and sub‐themes was reviewed, until team consensus was achieved. Next, associations and dissociations occurring amongst thematic content were identified. Finally, a visual representation of organizing and sub‐themes was generated. This analysis procedure was then applied to Chapter 11 of the CoP (2007).

### 2.3 Triangulation

Validation of findings involved: (a) comparing the emergent hierarchy of themes for the HRA research guidance documents and the CoP (2007) for points of corroboration; (b) Comparing points of corroboration with the summary of word referent frequencies.

## 3 RESULTS

### 3.1 Word referent frequency

Table 1 summarizes word referent frequency in the three domains of interest: people with communication and understanding difficulties; decision‐making and capacity; and inclusion in research. The total word referents per domain and the number of sources in which they occurred are shown (see Appendix A for document title). Each domain contains word referents as indicated in the central column. A ‘+’ immediately after the core referent indicates that it is a ‘multi‐stem’ phrase, to include related terms. For example, autism + included a variety of different words used to describe autism, including autistic spectrum disorder/condition/ASD/Asperger's syndrome/AS. Referents that did not include related terms are referred to in this paper as ‘single‐stem’ phrases. Referent frequency is rank ordered from highest to lowest within each category.

**Table 1:** Frequency of word referents (R) and number of sources (S)

Semantic Category	Word Referents	HRA documents: R (S)	CoP: R
People with communication and understanding difficulties	Aphasia+; autism+; attention+; dementia+; brain injury+; Brain disturbance; intellectual+; mental health+; communication + disable+; impair+;	264 (14)	10
MCA	Capacity+	396 (9)	36
	Consultee	122 (3)	5
	Cognition+	95 (12)	0
	Assent	21 (3)	0
	Decision‐making	9 (2)	0
Inclusion in research	Risk+	532 (11)	7
	Benefit+	365 (10)	10
	Protect+	158 (14)	3
	Equality	55 (8)	0
	Accessibility+	39 (8)	0
	Autonomy	20 (5)	0
	Inclusion	16 (6)	0
	Enable+	9 (4)	0
	Empower+	2 (2)	0
Media	Easy Read	63 (1)	0
	Audio	9 (4)	0
	DVD+	7 (2)	0

People with communication and understanding difficulties included a range of conditions, which is presented as a composite number of word referents (n = 264). The category ‘inclusion in research’ had the highest frequency overall, with *risk* dominating (n = 532), followed by *benefit* (n = 365) and *protect+* (n = 158) with the latter featuring in all 14 sources. In contrast, the combined referents *equality, accessibility+, autonomy, inclusion, enable + and empower+,* yielded a frequency of 141. ‘MCA (2005)’ contained the second most frequent word referents (n = 643) with *capacity + *dominating (n = 396), followed by *consultee* (n = 122), which referred to the recommended procedure for people lacking capacity. The combined frequency of *assent* and *decision‐making* was relatively small (n = 30). The referent ‘easy read’ (n = 63) was four times higher than the other media types, for example audio, DVD+, combined (n = 16).

### 3.2 Summative content analysis

**FIGURE 1:**
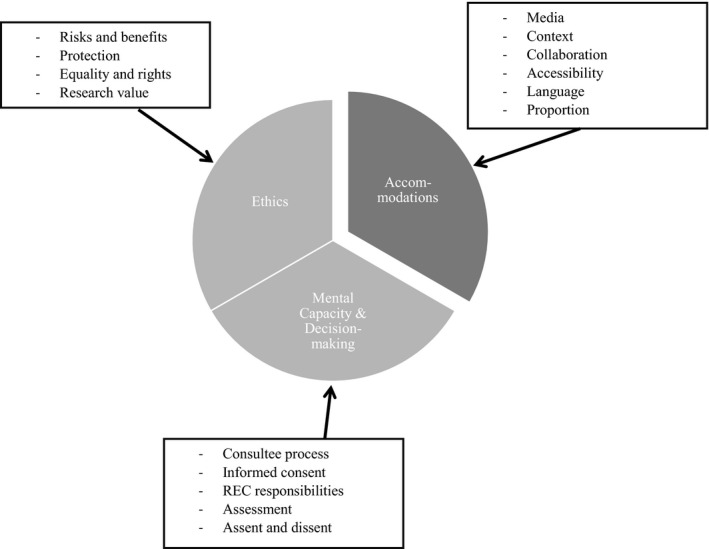
Schematic diagram of the three organizing themes and their sub‐themes

Initially, research guidance was defined by two levels of relevance: general (sources: 11, references: 327); and specific (sources: 14, references: 479). The first level, general, was excluded because of its focus on operational principles, rules and regulations associated with ethically sound research. The second level, specific, was included because it focused on the needs of people with communication and understanding difficulties. Three organizing themes emerged: *Ethics*; *Capacity & Decision‐making*; and *Accommodations* (Figure 1.) The first two organizing themes appeared to be interlinked, with *Ethics* focusing on the moral principles governing actions and decisions in relation to research, and *Capacity & Decision‐making* describing the enactment procedures. The third organizing theme, *Accommodations*, focused on considerations for people with communication and understanding difficulties participating in research.

Table 2 summarizes the content analysis of the research guidance and the CoP (2007). *‘Mental capacity & decision‐making’* occupied the greatest content in the research guidance (n = 206 references; 43%). The other two organizing themes were similar: *‘Ethics’* (n = 135; 28%) and *‘Accommodations’* (n = 138; 29%). For the CoP (2007), *‘Ethics’* (n = 51 references; 64%) represented the major content, which was above *‘Mental capacity & decision‐making’* (n = 28 references; 35%), with *‘Accommodations’* rarely mentioned (1%).

**Table 2:** Summary of organizing themes, sub‐themes and number (percentage) of references for the research guidance documents and the CoP (2007) arising from summative content analysis

Organizing themes	Sub‐themes	Policy guidance: n *(%)*	CoP: n (%)
Ethics	Equality and rights	34 (7%)	9 (11%)
	Protection	45 (9%)	13 (16%)
	Research value	14 (3%)	18 (23%)
	Risks and benefits	42 (9%)	11 (14%)
Mental capacity & decision‐making	Assent and dissent	18 (4%)	5 (6%)
	Assessment	25 (5%)	1 (1%)
	Consultee process	77 (16%)	12 (15%)
	Informed consent	50 (10%)	2 (3%)
	REC responsibilities	36 8%)	8 (10%)
Accommodations	Accessibility	13 (3%)	1 (1%)
	Collaboration	23 (8%)	0
	Context	27 (6%)	0
	Language	13 (3%)	0
	Media	52 (11%)	0
	Proportion	10 (2%)	0

#### 3.2.1 Ethics

*Ethics* featured in 11 research guidance documents (135 references). Four sub‐themes emerged, stated in descending order of reference frequency: ‘risks & benefits’ (n = 45) ‘protection’ (n = 42); ‘equality & rights’ (n = 34); and ‘research value’ (n = 14). The first two sub‐themes were connected to the well‐being and safety of people deemed to be vulnerable. Various factors were acknowledged to affect an individual, including socio‐economic status and educational level:

‘…highly dependent on the context. For example, people who are illiterate, marginalized by virtue of their social status or behaviour, or living in an authoritarian environment, may have multiple factors that make them vulnerable.’ [Source 2]

Although benefits were considered under ‘risks & benefits’ (n = 6 references), perceived threats dominated (n = 21 references). The sub‐themes ‘equality & rights’ and ‘research value’ focused on research participation opportunities being made available to all people, regardless of capacity. The importance of research being relevant to people was also highlighted:

‘…ethical medical research can fairly be regarded as “necessary in a democratic society in the interests of…the economic well‐being of the country” or of “the protection of health”, depending on the nature of the research.’ [Source 3]

Benefits of participation were discussed in connection to incapacity: inability of a participant to give informed consent implied that participation could only happen if direct benefits were apparent:

‘research without consent from a person should normally only occur if the research activity is considered to provide direct benefit to that person.’ [Source 1]

The safeguarding of participants was emphasized:

‘If someone is unable to provide consent for themselves due to a lack of mental capacity, the next step to consider is whether the legal requirements and safeguards can be met…alternatively the researchers should consider not including the person in question in the research.’ [Source 6]

whilst also ensuring equality of participation opportunities, particularly for groups under‐represented in research:

‘Groups that are underrepresented in medical research should be provided appropriate access to participation in research’. [Source 14]

For the CoP (2007), ‘Research value’ yielded the highest number of references (n = 18), which inferred that the research aims should be consistent with the impairing condition of the recruited participant as to be relevant:

‘The aim of the research must be to provide knowledge about the cause of, or treatment or care of people with, the same impairing condition – or a similar condition.’ [CoP: 11.12]

However, the CoP (2007) also asserted the importance of the protection of vulnerable people, particularly those individuals deemed to lack capacity:

‘…nothing must be done to or in relation to the person who lacks capacity which is unduly invasive or restrictive’. [CoP: 11.12]

and that the research benefits to the person who lacks capacity, be proportionate to any burden resulting from participation:

‘The research must have some chance of benefiting the person who lacks capacity…the benefit must be in proportion to any burden caused by taking part…’ [CoP: 11.12]

#### 3.2.2 MCA (2005) & Decision‐making

The *Mental Capacity & Decision‐making* organizing theme featured in 9 research guidance documents (206 references) where content was defined in 5 sub‐themes, stated in descending order of reference frequency: ‘consultee process’ (n = 77); ‘informed consent’ (n = 50); ‘REC responsibilities’ (n = 36); ‘assessment’ (n = 25); ‘assent & dissent’ (n = 18). Both ‘consultee process’ and ‘informed consent’ asserted the non‐coercive recruitment of participants:

‘When consent has been obtained orally, researchers should provide to the research ethics committee documentation of consent, certified either by the person obtaining consent or by a witness at the time consent is obtained’. [Source 2]

‘…respect requires giving them the opportunity to choose to the extent they are able, whether or not to participate in research’. [Source 13]

Similarly, except for ‘informed consent’ (n = 2), the 28 references identified in the CoP (2007) were primarily focused on: ‘consultee process’ (n = 12); ‘REC responsibilities’ (n = 8):

‘…arrangements to consult carers and to follow the other requirements of the Act’ [CoP: 11.11]

‘REC responsibilities’ referred to the duties carried out by the REC, identifying the checks and balances considered relevant to ensure ethical conduct of research and was evident in both the research guidance and the CoP (2007) (n = 8):

‘…must be satisfied that the research meets the relevant requirements relating to the nature, risks and benefits of the research and the arrangements in place to meet the other safeguards in the Act’. [Source 4]

‘Assessment’ focused on the principles of determining an individual's capacity for the purpose of research participation. This was mentioned mainly in the research guidance (25 references) and only once in the CoP (2007):

‘Mental capacity is considered to be lacking if, in a specific circumstance, a person is unable to make a decision for him or herself because of an impairment or a disturbance in the functioning of their mind or brain’. [Source 11]

‘Researchers should assume that a person has capacity, unless there is proof that they lack capacity to make a specific decision’. [CoP: 11.4]

The final sub‐theme of ‘assent & dissent’ acknowledged the diverse ways individuals may express their wishes, feelings and responses to research activities. In relation to incapacitous participants, the need to monitor assent or dissent, and to ensure such communicative signals are responded to appropriately was asserted in the research guidance (18):

‘…their assent should be regularly monitored by sensitive attention to any signs, verbal or non‐verbal, that they are not wholly willing to continue with the data collection’. [Source 1]

However, the requirement to be sensitive to the participant's responses was only inferred in the CoP (2007) (5 references):

‘…respect any objections a person who lacks capacity makes during research’. [CoP: 11.9]

#### 3.2.3 Accommodations

The *Accommodations* organizing theme featured in 10 research guidance documents (138 references) with only one reference made in the CoP (2007). This theme captured measures recommended for use with people who have communication and understanding difficulties. Centred mainly on the informed consent procedure, it included guiding principles and suggested modifications to activities and resources. Content was defined in six sub‐themes, stated in descending order of reference frequency: ‘media’ (n = 52); ‘context’ (n = 27); ‘collaboration’ (n = 23); ‘accessibility’ (n = 13); ‘language’ (n = 13); ‘proportion’ (n = 10); ‘Media’ focused on the presentational formats that might be used to deliver information, where Easy Read (a familiar format used particularly by people with intellectual disabilities—usually characterized by simple language accompanied with associated pictorial images) dominated (n = 18), although, pictures, manual sign, moving images and audio were also referenced:

‘Easy Read is not the only way to communicate with people with learning disabilities. Other methods include video, talks, presentations, drama, murals, role‐play or posters’. [Source 5]

‘Collaboration’ considered public involvement in the development of research materials, for example, information sheets, and emphasized the importance of coproduction. It identified the important role of the expert‐by‐experience in guiding how information is presented:

‘It’s about working with people your information is for, finding out together how you can make the information useful and accessible for them’. [Source 5]

‘Accessibility’ and ‘proportion’ established the importance of modifying project information and associated procedures, to support understanding about the research. These areas captured the key principles of rendering information about research in a way that would be meaningful:

‘…establish whether informed consent could be modified in a way that would preserve the participant’s ability to understand the general nature of the investigation...’ [Source 2]

and manageable to people with capacity difficulties:

‘…application of the principle of proportionality to the provision of information to potential research participants’ [source 7]

‘…provide this information in a succinct way which provides the core detail that participants need to know in a meaningful fashion without overloading’ [Source 7]

Only one oblique reference was made to the need for support in the CoP (2007):

‘…the person must also receive support to try to help them make their own decision…’ [CoP: 11.4]

The sub‐theme ‘context’ covered factors associated with the presentation and delivery of information to potential participants, including person‐centred considerations, dialogue and time. Respectively, the need to tailor materials and procedures to the individual, and the importance of providing opportunities to interact about the research:

‘Interactive questioning of potential participants within the consent process can aid their understanding of the information presented’. [Source 7]

and the availability of time for processing information was acknowledged:

‘Each individual must be given as much time as needed to reach a decision, including time for consultation with family members or others’. [Source 2]

The sub‐theme of ‘language’ focused largely on the form, or the grammatical properties of the communication, with a more limited appraisal of vocabulary and meanings, and the intended purpose or function of the communication:

‘The language should be clear and accessible to people with limited literacy, using short words and sentences, written in the active voice, and avoiding the use of technical terms’. [Source 1]

### 3.3 Triangulation

The organizing themes of the research guidance and the CoP (2007) had variable emphases. Whilst ‘mental capacity & decision‐making’ was dominant in the research guidance, ‘ethics’ occupied the greater content of the CoP (2007). Regardless of relative weighting, ‘ethics’ and ‘mental capacity & decision‐making’ were interconnected. That is, both organizing themes corresponded to governance procedures under the MCA (2005). The importance of risk management was evidenced in references to ‘protection’ and ‘risks & benefits’ in both the research guidance and the CoP (2007), with the latter promoting the importance of ‘research value’. Excluding multi‐stem phrases, the top three single‐stem word referents identified within the research guidance and CoP (2007) were capacity, risk and benefit (see Table 1). In the HRA documents, ‘Accommodations’, whilst occupying 138 references, were presented as isolated position statements that did not appear to connect to the other two organizing themes. Content was restricted to choice of media for delivering information (n = 59) with ‘easy read’ dominating (n = 18). This reflects the frequency in the key word referents used in the documents (Easy Read = 63; audio = 9; DVD+ =7). The CoP (2007) contained just one generic statement on the need for support. Thus ‘accommodations’ appeared to be additional to the major organizing themes in both the HRA documents and the CoP (2007).

## 4 DISCUSSION

Protection of individual rights under the law through the relevant governance procedures formed the major content of the research guidance and the CoP (2007), consistent with previous work by Heywood and colleagues.^22^ The CoP (2007) elaborated on the MCA (2005) principles by considering ‘research value’ as critical to the protection of incapacitous individuals, and as justification for their involvement in research.

Conversely, empowerment was addressed infrequently within the research guidance and the CoP (2007), which is consistent with comments from the House of Lords select committee.^2^ In accordance with the legal test for capacity, the research guidance adopted a binary approach to mental capacity assessment, whereby a person was either assessed to have capacity or not.^21^, ^22^, ^29^, ^30^ However, fluctuations in capacity and how these affect an individual's eligibility to participate in research requires consideration.^21^, ^22^, ^29^, ^30^ Although the need for accommodations was recognized, detail into how they might serve the principles of determining and supporting an individual's capacity and communication was not provided.^22^ Thus, the idea of joining efforts with others in order to achieve understanding, which is consistent with empowerment theory,^35^ was largely neglected. The inclusion of incapacitous participants in research was linked to procedural aspects of the consultee process, with limited practical strategies for facilitating engagement and decision‐making that is evidenced in the emerging use of assent in some areas of research, for example dementia.^6^, ^44^, ^56^ More generally, within the research guidance there was encouragement for providing a supportive context for delivering information by considering time, place and the development of dialogue with the individual. This suggests a more collaborative enterprise to information presentation^37^ where setting factors are considered in the promotion of individual understanding. However, the process whereby meanings are constructed was neglected.^48^

The connection between protection and empowerment of people with communication and understanding difficulties might possibly be served by accommodations, which enable optimal understanding by participants whatever their capacity. Although the use of multiple media for presenting information was considered in the research guidance, such as Easy Read for people with intellectual disabilities, little attention was given to the critical aspects of language.^51^ Accordingly, advice provided on reasonable adjustments and practical ways of conveying information targeted surface‐level features, for example syntax, and neglected deeper level features of semantics (construction of meaning) and use (the intended effect of the communication).^52^ Similarly, within the CoP (2007) there was little mention of accommodations to support communication and there were no practical examples of how to effectively use accommodations. Although there was mention of capacity assessment within the CoP (2007) in relation to decisions to take part in research, practical guidance to support researchers in this endeavour was absent. Furthermore, it neglected to discuss accommodations to support the assessment of capacity. This lack of practical guidance within the CoP could explain why it has been poorly implemented.^23^

The current study was restricted to analysis of primary sources that were publicly available on the HRA website. It did not extend to other resources published by organisations outside the HRA, supplementary to the MCA (2005) and CoP (2007). Investigation into access and usage of the sources would have provided insights into the uptake of the guidance, but this was not explored in the current study. Examination of key word referents in the documents was a surface level, textual analysis—limited to frequency of vocabulary items. However, it provided a useful precursor to the more detailed summative content analysis where meanings were explored.

In conclusion, the CoP (2007) and research guidance provided definitions for some of the technical aspects of the MCA and therefore could be considered to support the representation of people with communication and understanding difficulties in research in England and Wales. However, the emphasis was on formal governance procedures related to protection and risk management. Furthermore, the constructs of protection and empowerment appeared to be somewhat polarized, with the relationship between the two being an underdeveloped concept. Therefore, the question of how to empower potentially vulnerable research participants, whilst also providing adequate protection, remains. This points to a strong and enduring need for guidance that focuses on the researcher's efforts to promote the autonomy of the participant as far as possible, regardless of their assessed capacity. This includes skilful use of language for meaning construction and communication strategies for information retention and processing; deliberately addressing setting factors for promoting understanding and recall; and the use of a variety of media for representing and conveying information. These strategic accommodations might serve to mediate the apparent disconnect between protection and empowerment, enabling people with communication and understanding difficulties to have a voice in research.

## CONFLICT OF INTEREST

The authors declare that they have no competing interests. All authors listed on the manuscript contributed to the study design, data collection, data analysis and interpretation of the findings, and drafting and reviewing the final manuscript.

## AUTHOR CONTRIBUTIONS

HR was involved in data retrieval, analysis, interpretation and visualisation; writing of the original draft and review and revision of subsequent drafts. RH was a consultant on all legal aspects of the work and review of the original draft and contributing to subsequent drafts. OJ, AK, PEL and CS were involved in review of the original draft and contributing to subsequent drafts. KB was involved in the design, data analysis and interpretation; writing of the original draft and review and revision of subsequent drafts.

## Data Availability

The data addressed in this paper are publicly available via the UK Health Research Authority website (www.hra.nhs.uk).

## APPENDIX A

**Table A1:** Summary table of HRA research guidance documents including in sample

	Title	Year	Author
1.	Code of Human Research Ethics.	2014	British Psychological Society (BPS)
2.	International Ethical Guidelines for Health‐related Research Involving Humans.	2016	Council for International Organisations of Medical Sciences (CIOMS)
3.	Mental Capacity Act 2005 and consent for research.	No date given	Department of Health
4.	Guidance on nominating a consultee for research involving adults who lack capacity to consent.	2008	DH Scientific Development and Bioethics Division
5.	Making written information easier to understand for people with learning disabilities.	2010	Department of Health
6.	Care after research: A framework for NHS RECs.	No date given	Health Research Authority
7.	Applying a proportionate approach to the process of seeking consent.	2017	Health Research Authority
8.	Policy and Procedure for the Recruitment and Selection of Members of Research Ethics Committees including Reappointment, Break in Service, Probationary Periods, Leavers and Transfers.	2008	Health Research Authority
9.	Impact of public involvement on the ethical aspects of research.	2016	Health Research Authority
10.	Public involvement in research and research ethics committee review.	2016	Health Research Authority
11.	MRC Ethics Guide. Medical research involving adults who cannot consent.	No date given	Medical Research Council
12.	MRC Guidance on managing risk in public health research.	No date given	Medical Research Council
13.	The Belmont Report	1979	National Commission for the Protection of Human Subjects of Biomedical and Behavioural Research
14.	World Medical Association Declaration of Helsinki. Ethical Principles for Medical Research Involving Human Subjects.	2013	World Medical Association

**Table A2:** Summary table of key word referents (stem + expansions and related terms)

People with communication and understanding difficulties	Aphasia+ (aphasic; dysphasic; acquired language disorder/impairment) Autism+ (spectrum disorder/condition; autistic; Asperger/’s) Attention+ (deficit; hyperactivity; disorder) Dementia+ (Alzheimer's) Acquired Brain injury+ (head injury) Brain disturbance Intellectual+ (learning) Mental health+ (condition/s; difficulty/s; disorder/s; issue/s; problem/s; ill‐health, mental illness, psychological well‐being) Communication+ (support; aid/s; strategy/s; tool/s) Disable+ (disability/s; disabled) Impair+ (‐ed; ‐ment; deficit; difficulty; problem; disorder)
MCA (2005)	Capacity+ (incapacity)
	Consultee
	Cognition+ (cognitive)
	Assent
	Decision‐making
Inclusion in research	Risk+ (‐s)
	Benefit+ (‐s; ‐ed)
	Protect+ (‐ion; ‐ing)
	Equality
	Accessible+ (access/ibility)
	Autonomy
	Inclusion
	Enable+ (‐ed; support)
	Empower+ (‐ment; ‐ing)
Media	Easy Read
	Audio
	DVD (Video)
